# Cellulose Acetate Membranes Modification by Aminosilane Grafting in Supercritical Carbon Dioxide towards Antibiofilm Properties

**DOI:** 10.3390/membranes12010033

**Published:** 2021-12-27

**Authors:** Marcin Tyrka, Mariusz Nowak, Dusan Misic, Tomasz Półbrat, Stanisław Koter, Anna Trusek, Irena Zizovic

**Affiliations:** 1 Department of Bioprocess, Micro and Nano Engineering, Wroclaw University of Science and Technology, Wybrzeze Wyspianskiego 27, 50-370 Wroclaw, Poland; marcin.tyrka@pwr.edu.pl (M.T.); mariusz.nowak@pwr.edu.pl (M.N.); anna.trusek@pwr.edu.pl (A.T.); 2 Department of Functional Foods Development, Wroclaw University of Environmental and Life Sciences, Chelmonskiego Street 37, 51-630 Wroclaw, Poland; dusan.misic@upwr.edu.pl (D.M.); tomasz.polbrat@upwr.edu.pl (T.P.); 3 Department of Physical Chemistry and Physicochemistry of Polymers, Nicolaus Copernicus University in Torun, Gagarina 11 Street, 87-100 Torun, Poland; skoter@umk.pl

**Keywords:** cellulose acetate, membrane, antibiofilm, aminosilane, supercritical carbon dioxide, grafting

## Abstract

The study explores the grafting of cellulose acetate microfiltration membranes with an aminosilane to attain antibiofilm properties. The grafting reaction was performed in the supercritical carbon dioxide used as a transport and reaction medium. The FTIR analyses and dissolution tests confirmed the covalent bonding between the aminosilane and polymer. The membranes’ microstructure was investigated using a dual-beam SEM and ion microscopy, and no adverse effects of the processing were found. The modified membranes showed a more hydrophilic nature and larger water permeate flow rate than the neat cellulose acetate membranes. The tests in a cross-filtration unit showed that modified membranes were considerably less blocked after a week of exposure to *Staphylococcus aureus* and *Escherichia coli* than the original ones. Microbiological investigations revealed strong antibiofilm properties of the grafted membranes in experiments with *Staphylococcus aureus*, *Listeria monocytogenes*, *Escherichia coli*, and *Salmonella* Enteritidis.

## 1. Introduction

One of the main concerns in membrane applications, whether in water and wastewater management, bioreactors, the biomedical field, or other applications, is biofouling [1,2]. Adhesion of microorganisms onto the membrane surface, followed by biofilm production, poses additional resistance to mass transfer in the filtration process, requiring a larger driving force to maintain productivity [1,3]. In addition, in biofilm, microorganisms are protected from disinfectants and antimicrobials by self-produced extracellular polymeric matrices, and consequently, they are difficult to remove [1,2,4]. Microorganisms’ deposition and multiplication also present a significant issue in membrane filters used for air conditioning. Different ways of membrane surface modification were proposed to overcome this problem, including grafting, deposition of metal ions and nanoparticles, polymer blending, coating, and incorporation of antimicrobial substances into the polymer matrix [1,3,5].

Grafting implies a chemical modification, e.g., covalent bonding between a substance providing the desired activity and the solid phase [4,6,7]. A review of Lee et al. [7] summarized grafting techniques and membrane-monomer systems, recognizing specific grafting techniques such as photo-induced grafting, plasma treatment and plasma-induced grafting, radiation-induced grafting, thermal-induced, and ozone-induced grafting [7]. Generally, in the reported studies, monomer grafting was performed to reduce the interaction between undesirable solutes and particulates with the surface, introduce suitable charged groups to create electrostatic repulsion from the membrane surface, or improve hydrophilicity for better surface-water interaction [7,8,9]. There are several reports on using ethoxy or methoxy silanes for grafting purposes, where hydroxyl groups at the surface of the solid phase were needed for the grafting reaction [10,11,12]. He et al. reported bacterial cellulose grafting by 3-aminopropyltriethoxysilane as a method to produce membranes with antibiofilm properties for biomedical applications [10]. The grafting was performed by stirring the membranes in the aminosilane solution at room temperature for four hours [10]. In another study, Achoundong et al. [11] successfully modified cellulose acetate dense films with vinyltrimethoxysilane to obtain membranes for acid gas removal. The reaction was performed at 200 °C for 24 h [11]. 

Due to their specific properties, supercritical fluids have been increasingly applied in many areas from separations to material design, impregnation, biomass treatment, and energy production, but not limited to the aforementioned [13,14,15]. High density, low viscosity, high diffusion coefficients, and absence of surface tension in the supercritical phase allow easy penetration in solid matrices. The supercritical fluid can act as a solvent, plasticizer, transport medium for an active component, or reaction medium. Moreover, the properties of supercritical fluids (density, e.g., solvating power, diffusivity coefficient) may be easily tuned by changing the pressure or temperature. The most applied supercritical fluid, due to its favorable critical temperature (31.1 °C), nontoxicity, inflammability, availability, and low price, is carbon dioxide. Supercritical carbon dioxide (scCO_2_) is considered to be a green solvent [16,17] and an efficient tool in material processing and design where the elimination of traditional waste generation is also possible (e.g., wood impregnation, textile dyeing) [18,19,20]. Since carbon dioxide is a gas under atmospheric conditions, its separation from the solid matrix is easy and complete, simply by the pressure reduction. Thus, a product free of solvent residues is obtained [19,21]. An additional advantage of applying scCO_2_ in materials design is the possibility of processing finished polymeric forms. The last option brought solutions to the production of hip and knee endoprosthesis [22,23] and ophthalmological contact lenses [24,25]. Another significant advantage provided by supercritical fluids compared to other techniques is the modification of the solid phase throughout the whole volume due to the absence of surface tension [22,23]. The mentioned favorable properties of scCO_2_ have recently been exploited for grafting purposes as well. Xu et al. [26] chemically attached antibacterial quaternary ammonium compounds to hydroxyl groups of cellulose, hemicellulose, and lignin in softwood via hexamethylene diisocyanate as a linker. Darpentigny et al., in their recent study [12], presented the feasibility of cellulose nanopapers grafting with an aminosilane in scCO_2_. The product obtained showed antibacterial properties. 

Our previous studies demonstrated that it was possible to modify commercial polyamide and cellulose acetate microfiltration membranes by impregnation in scCO_2_ with thymol and carvacrol as antibacterial agents [4,27,28]_._ The active substance loadings over 30% were possible without membranes microstructure disturbance. Thymol and carvacrol were attached to the polymer matrix by hydrogen bonding, and modified membranes showed strong antibiofilm properties [4,27]. However, though strong, the membranes’ activity was time-limited due to the high volatility of active compounds.

In this study, we examine a possibility of an active substance covalent bonding to the membranes’ polymer matrix in scCO_2_ to obtain antibiofilm properties. Commercial cellulose acetate membranes and 3-aminopropyl (diethoxy)methylsilane (APDEMS) were selected as the membrane and active substance models to test this environmentally friendly approach in added value membranes formulation. The modified membranes were investigated by the FTIR method, contact angle measurements, porosimetry, and dual-beam SEM–FBI microscopy and tested in a cross-filtration unit. The evaluation of bacterial adhesion was performed with *Staphylococcus aureus*, *Listeria monocytogenes*, *Escherichia coli*, and *Salmonella* Enteritidis.

## 2. Materials and Methods

### 2.1. Materials

Commercial cellulose acetate (CA) microfiltration membranes with 0.2 µm average pore diameter and 47 mm membrane diameter were supplied by GE Healthcare Whatman TM, Japan (Cat. No. 7001-0004). CA in the form of beads, Eastman CA-320S, was a generous donation from Safic-Alcan Poland. CA powder (Mn ~30,000), 3-aminopropyl (diethoxy)methylsilane (APDEMS), and toluene (≥99.5%) were purchased from Sigma-Aldrich, Germany. Acetone p.a. was provided by Stanlab (Lublin, Poland). Carbon dioxide (purity > 99.99%) was supplied by Air Liquid, Wrocław, Poland.

### 2.2. Grafting Reaction in Supercritical Carbon Dioxide

**Figure A1:**
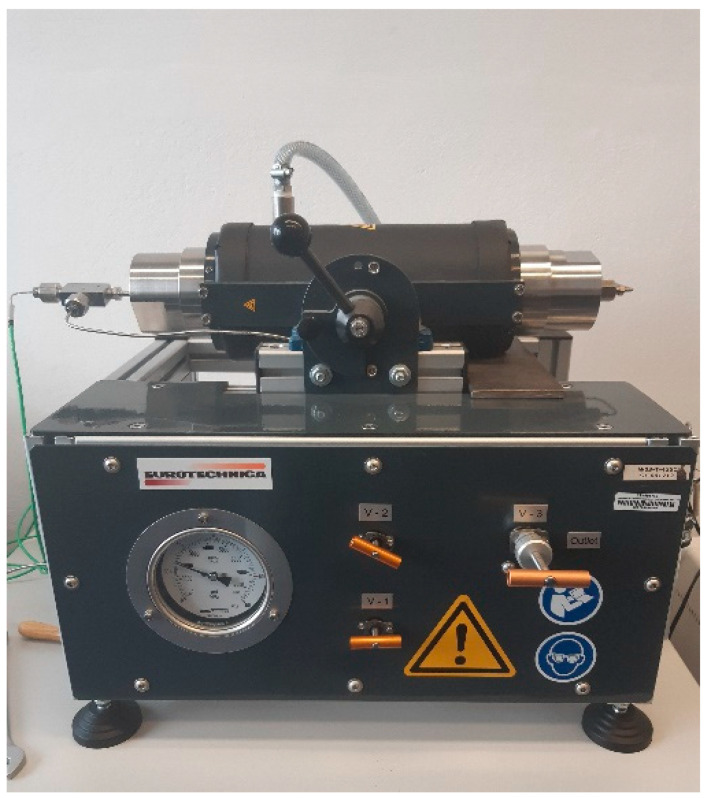
Tilting high-pressure vessel (280 mL, Eurotechnica GmbH, Germany).

Commercial CA membranes were treated with APDEMS in a 280 mL volume high-pressure vessel (Eurotechnica GmbH, Bargteheide, Germany) presented in Figure A1 (Appendix A), equipped with a heating jacket, where water was used as the heating fluid. A heating bath circulator (Jeio Tech Co., Ltd., Daejeon, Korea) was used to recirculate water and maintain its temperature. Membranes were exposed to water vapor for 10 s before the experiment to ensure sufficient humidity. Three membranes were placed in the vessel. Next, 3 mL of 1:1 vol. mixture of APDEMS and toluene were placed in a glass vessel between every two membranes (6 mL of the mixture in total). Upon reaching the desired temperature, carbon dioxide was pumped into the system by an air-driven gas booster (Eurotechnica GmbH, Bargteheide, Germany). The reaction was performed at 50 °C and 12 MPa. The reaction pressure and temperature were adopted from the study of Darpentigny et al. [12]. The reaction time was investigated in preliminary experiments and adopted to be 12 h. A moderate decompression rate of 0.25 MPa/min was applied after 12 h of the contact time. The curing step (water elimination) followed. Darpentigny et al. [12] demonstrated the curing step efficiency in scCO_2._ The water elimination and establishment of covalent bonding were performed in scCO_2_ at 50 °C and 12 MPa. The membranes were exposed to supercritical fluid in a batch mode for 1.5 h. After that, a 2 L/min scCO_2_ flow rate was established through the vessel for another 30 min. The latter mode was used to ensure the elimination of unreacted APDEMS traces in the membranes. 

The membranes’ mass change was followed by an analytical balance, and the grafting yield (*Y*) was calculated as

(1) $$Y=\frac{W-W_{0}}{W_{0}}\cdot 100\%$$

where *W* is the mass of grafted membrane, and *W*_0_ is the mass of the initial untreated membrane. 

Several grafted membranes were subjected to a dissolution test. They were stirred in toluene for 5 min to evaluate if any free silanes remained in the polymer matrix. 

Grafting of cellulose acetate in the form of beads and films was also performed for the FTIR and contact angle analyses. The cellulose acetate films were produced by the solvent casting method described in Appendix A. 

### 2.3. FTIR Analyses

The Fourier-transform infrared (FTIR) spectroscopy analysis was performed for chemical characterization. The spectra of the neat and grafted CA membranes and beads were recorded in the ATR mode using Nicolet iS50 Spectrometer (Thermo Fisher SCIENTIFIC) with a resolution of 4 cm^−1^ at wavenumbers in the range of 500–4000 cm^−1^.

### 2.4. Structural Properties Investigation 

The structural properties of neat and grafted membranes were investigated using a two-beam microscope SEM/Ga-FIB FEI Helios NanoLab™ 600i, which comprises ultra-high resolution electron and ion microscopy. An energy focused beam of gallium ions provides the ability to perform the sample cross-sections by the selective removal of the preparation material and modification at the nanoscale. Before the analyses, the samples were coated with gold. Coulter^®^ Porometer II (Coulter Electronics Ltd., Luton Bedfordshire, UK) with porofil as a wetting liquid was used to determine the pore size distribution in the neat and grafted membranes.

### 2.5. Contact Angle Measurements 

A goniometer model OCA 15EC (DataPhysics, Filderstadt, Germany) was used to investigate the water contact angle of neat and grafted CA films. DataPhysics’ picolitre dosing system (PDDS) enables to reproducibly dose droplets of down to 30 picolitres. The water drop was recorded by a camera, and the contact angle was analyzed upon the contact and after 3, 6, 9, and 12 s of the contact time.

### 2.6. Test in a Cross-Filtration Unit 

A laboratory cross-filtration unit described in detail elsewhere [4,27] was used to investigate the water permeation flux through the neat and grafted membranes and evaluate the modification impact on membrane functionality. The cross-filtration unit was also used to assess the membranes’ blockage due to exposure to bacterial cells. The grafted and neat membranes were incubated under the static conditions for one week at 37 °C in the presence of *Staphylococcus (S.) aureus* (volume 150 mL, initial cell concentration 14.5 × 10^6^ CFU/mL) and *Escherichia (E.) coli* culture (volume 150 mL initial cell concentration 14.8 × 10^6^ CFU/mL). After the incubation period, the membranes were rinsed twice with distilled water, and the permeate flow rates of water were recorded for different transmembrane pressures. For the comparison, the water permeate flow rates through the membranes were also measured before exposure to bacteria.

*S. aureus* DSM 2569 and *E. coli* DSM 4509 were cultured in 500 mL flasks in 160 mL of an appropriate medium. Each medium was inoculated with 10 mL of 48 h-old culture and incubated at 37 °C at 180 rpm (IKA KS 4000). The medium for *S. aureus* contained (in grams per liter): casein hydrolysate 10, thiamine 0.26, nicotinamide 0.05, Na_2_HPO_4_ × 12 H_2_O 15.2, KH_2_PO_4_ 3.0, NaCl 0.5, NH_4_Cl 1.0, MgSO_4_·7H_2_O 0.25, and CaCl_2_ 0.01. The medium for *E. coli* included (in grams per liter): glucose 6.0, Na_2_HPO_4_·12H_2_O 15.2, KH_2_PO_4_ 3.0, NaCl 0.5, NH_4_Cl 1.0, MgSO_4_·7H_2_O 0.25, and CaCl_2_ 0.01. 

### 2.7. Investigations of Bacterial Adhesion to the Membranes

*S. aureus* ATCC 29213, *Listeria (L.) monocytogenes* ATCC 13932, *E. coli* ATCC 10536 and *Salmonella (S.)* Enteritidis (*Salmonella enterica* subspecies *enterica* serovar Enteritidis) ATCC 13076 (Microbiologics, St.Cloud, MN, USA) were used for the microbiological tests. Neat cellulose acetate membranes marked as CA and grafted membranes marked as gCA were cut into 1 cm^2^ squares and sterilized in an autoclave at 121 °C for 15 min. To evaluate the bacterial attachment to the polymer surface, the previously described method was applied [29]. In short, investigated strains were pre-incubated during 24 h at 37 °C in Cation adjusted Mueller Hinton broth (CAMHB, Becton Dickinson, Heidelberg, Germany) with the addition of 1% glucose (Merck, KGaA, Darmstadt, Germany) to stimulate exopolysaccharide production. After this, an initial bacterial inoculum of approximately 1–2 × 10^8^ CFU/mL (OD_550_ 0.10–0.12) has been prepared. Three tubes were filled with 2 mL of bacterial culture; the gCA sample was immersed in tube one, the CA sample was immersed in tube two, and the third tube served as a growth control without membrane samples. Two groups of test tubes were set up, group one was incubated for 24 h, and group two for 48 h, both groups at 37 °C. After incubation, the membrane samples were carefully removed from the broth with sterilized tweezers and washed gently with sterile Ringer’s solution (112 mM NaCl, Merck KGaA, Darmstadt, Germany; 6 mM KCl, Chempur, Piekary Śląskie, Poland; 2 mM CaCl_2_, Eurochem BGD, Tarnów, Poland; 1M NaHCO_3_, Eurochem BGD, Tarnów, Poland) to remove planktonic cells. The washed polymer samples were immersed in 10 mL of sterile Ringer’s solution and sonicated in an ultrasonic bath (37,000 Hz, Elmasonic S60, Elma Schmidbauer GmbH, Singen, Germany), 15 s/min, for a total of 5 min, to detach firmly adhered bacteria. Upon completion of the ultrasound treatment, five serial dilutions were performed (the first dilution 10^−1^ was the sonicated tube, up to 10^−6^) by taking 0.5 mL of the dilution being in contact with the membrane and transferring it to 4.5 mL of the Ringer’s solution. Each dilution was inoculated in three 10 μL aliquots on Tryptic soy agar (Becton Dickinson, Heidelberg, Germany), which were then incubated 24 h at 37 °C. Only replicates from two successive dilutions with no less than 15 and no more than 300 grown colonies were included in the calculation. The number of obtained CFU/mL was calculated according to the formula from the ISO standard [30]:

(2) $$NCFU=\frac{\sum C\;}{V\;\cdot \left[n1+0.1\cdot n2\right]\;\cdot \;d}$$

where ∑*C* is the sum of the colonies counted on all the replicates retained from two successive dilutions, *V* is the volume of inoculum applied to each Petri dish, in milliliters; *n*1 is the number of replicates retained at the first dilution; *n*2 is the number of replicates retained at the second dilution; *d* is the dilution factor corresponding to the first dilution.

The calculation of the number of attached bacteria per 1 cm^2^ (*NP*) was done by the formula:

(3) $$NP=\frac{NCFU\;\cdot \;V}{P}$$

where *NCFU* is the total number of detached bacteria in 1 mL of the medium, *V* is the volume of medium where the detachment has been performed, and *P* is the total surface in cm^2^ of the investigated material. Each test was performed three times.

## 3. Results and Discussion

### 3.1. Grafting in scCO_2_

The reaction conditions of 50 °C and 12 MPa were adopted from the study of Darpentigny et al. [12] on grafting cellulose nanopapers with an aminosilane in scCO_2_. The grafting time was investigated in preliminary experiments. A statistically valid number of FTIR analyses (10–15) was applied to each grafted membrane to evaluate its surface modification. Unreacted polymer regions could be detected in membranes after the contact time of several hours. A contact time of 12 h was found as appropriate to provide even surface modification detected by FTIR. 

The water elimination step (curing) was performed in scCO_2_ at 50 °C and 12 MPa for two hours. In the last 30 min of curing, a flow of scCO_2_ was provided to ensure extraction of possible unreacted APDEMS. The experiments were performed tenfold, and the grafting yield of 21% ± 2.2% was observed. Additionally, several grafted membranes were soaked in toluene, stirred for five minutes, and left for drying. After the toluene treatment, the membranes did not change in weight, proving that APDEMS was covalently bound to the polymer. There are no data in the literature about grafting in the supercritical phase for comparison. He et al. [10] reported grafting yields from 13.8% to 41.9% for bacterial cellulose and 3-aminopropyltriethoxysilane.

### 3.2. FTIR Analyses

**Figure 1:**
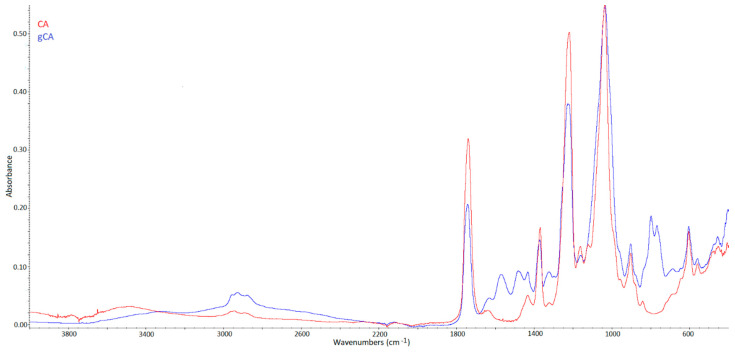
FTIR spectra of neat (CA) and grafted (gCA) membranes.

FTIR spectra of neat and grafted cellulose acetate membranes (CA and gCA, respectively) are presented in Figure 1. Characteristic peaks for cellulose acetate are visible in the spectrum of the neat CA membrane. The broad absorption band around 3470 cm^−1^ is ascribed to O-H stretching of the hydroxyl group [11,31,32]. The peak at 1741 cm^−1^ is assigned to the stretching of the C=O group [31,32,33]. The bands at 1433, 1367, and 1219 cm^−1^ originate from the C-H bending, rocking, and wagging vibrations, respectively [32,34]. The peak at 1036 cm^−1^ is assigned to C-O-C linkage in the glycosidic unit, and the peak at 901 cm^−1^ is a characteristic of saccharide [32,35,36,37].

**Figure 2:**
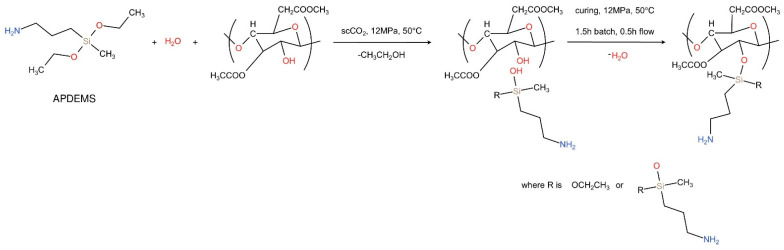
Proposed grafting reaction.

Proof that grafting occurred in our system is the disappearance of the broad absorption band at 3470 cm^−1^ (assigned to the hydroxyl group) in the spectrum of the grafted membrane. A similar conclusion was derived by Achoundong et al. [11]. The bands at 2934 and 2978 cm^−1^ are attributed to –CH_2_ and –CH_3_ asymmetric stretching vibrations [38], while a band appearing in the gCA spectrum around 1565 cm^−1^ is assigned to the N-H bending [10]. The new peaks around 1480 cm^−1^ originate from C-H bending in grafted aminosilane [38]. Bands around 1300 cm^−1^ are attributed to Si-CH_2_ and Si-CH_3_ stretching vibrations [38]. Being buried by the intense C–O–C vibration bands of cellulose acetate, typical signals of Si–O–C and possible Si–O–Si bridges, which should appear at about 1150 and 1135 cm^−1^ [10], could not be visible in the spectrum of the grafted membrane. In the spectrum of the modified membrane, the peaks indicating the presence of unreacted alkoxy groups of APDEMS appeared around 790–760 cm^−1^ [38]. A similar effect of the unreacted alkoxy groups’ presence after cellulose acetate films grafting with silane was reported by Achoundong et al. [11]. Based on these findings, the reaction mechanism presented in Figure 2 might be assumed. In the proposed scheme, R is the most likely ethoxy group. However, some ethoxy groups might have reacted further with other APDEMS molecules.

**Figure 3:**
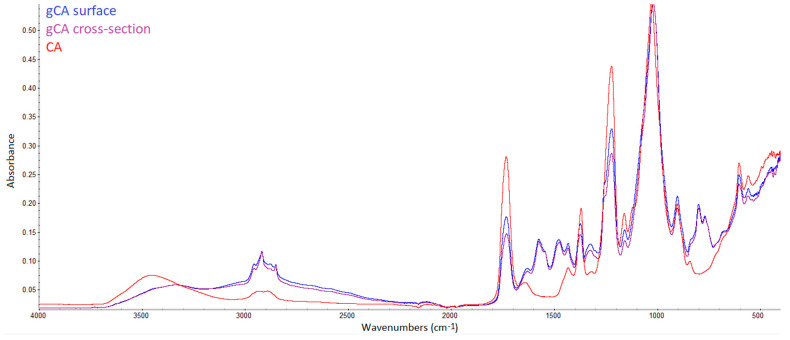
FTIR spectra of the neat bead (CA), and grafted bead’s (gCA) surface and cross-section.

The previously mentioned advantage of supercritical fluids application, which is the possibility of solid matrix modification through the whole volume, can be demonstrated using FTIR analysis. For that purpose, cellulose acetate beads were modified and cut. FTIR spectra of the neat bead and grafted bead surface and cross-section are presented in Figure 3. Very similar results were obtained for the surface and cross-section spectra. 

### 3.3. Structural Properties Investigation 

**Figure 4:**
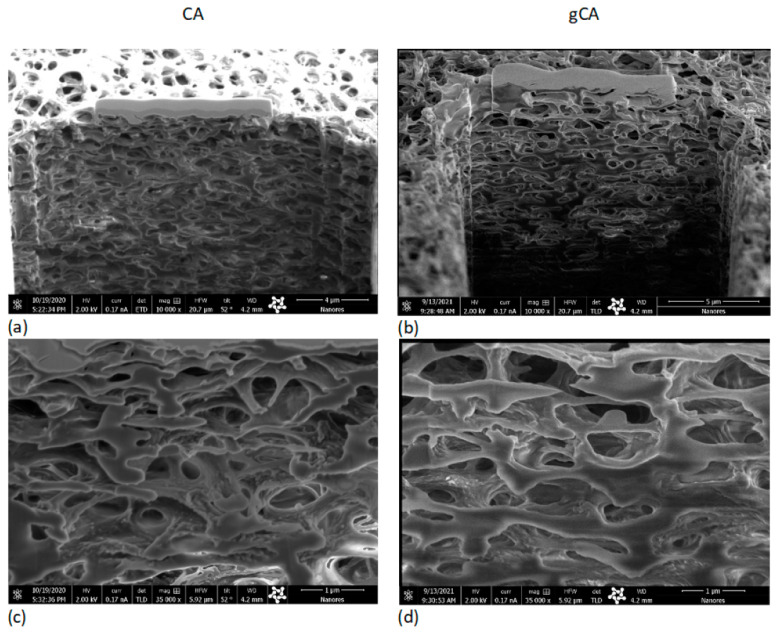
SEM images of the neat membrane (CA) and grafted membrane (gCA) cross-section (bar (**a**) = 4 µm; bar (**b**) = 5 µm; bar (**c**) = bar (**d**) = 1 µm).

**Figure 5:**
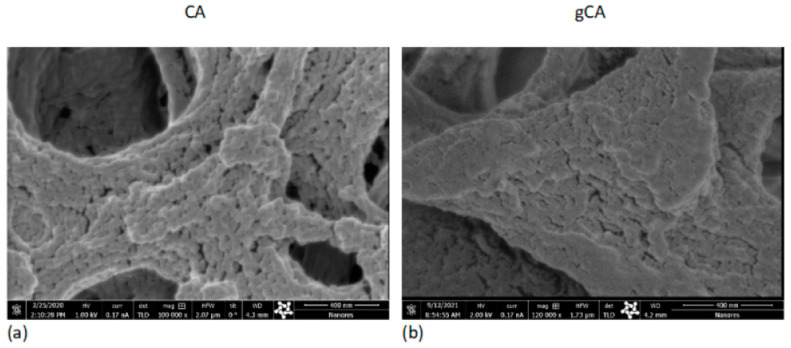
SEM images of the neat membrane (CA) and grafted membrane (gCA) surface (bar (**a**) = bar (**b**) = 400 nm).

The processing effect on the membranes’ microstructure has been investigated using the SEM–FIB method. Cross-sections of the neat (CA) and grafted membrane (gCA) are presented in Figure 4. No side effects of the high-pressure treatment to the membrane microstructure could be observed. SEM images obtained under larger magnifications (100,000–120,000×, bar = 400 nm) presented in Figure 5 reveal a change in a bump-like structure of virgin CA on the 50 nm scale. 

**Figure 6:**
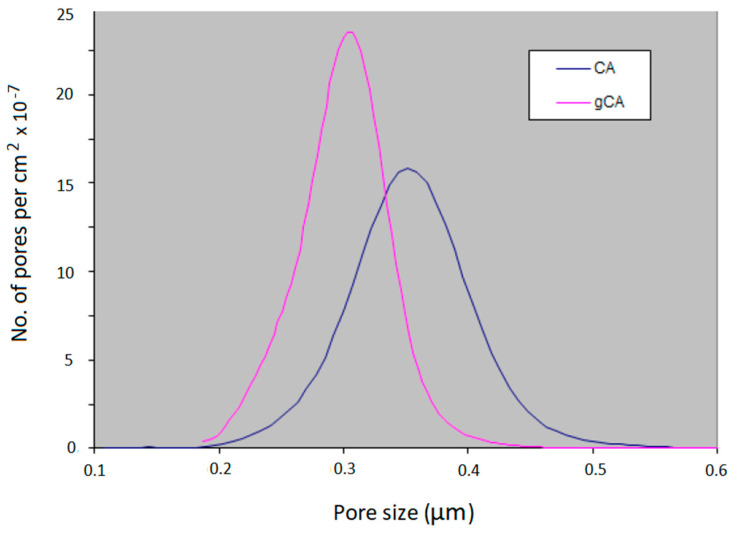
Pore size distribution in the neat and grafted cellulose acetate membrane.

Results of the pore size distribution analyses are presented in Figure 6. As can be seen, grafting caused a decrease in the mean pore size diameter from 0.374 µm to 0.321 µm. The grafted membrane showed a narrower pore size distribution (min 0.250 µm, max 0.358 µm) compared to the neat membrane (min 0.292 µm, max 0.494 µm). The modification induced a membrane porosity change from 71% to 67.3 %. It is important to stress that, due to the absence of surface tension in the supercritical phase, the polymer transformation occurred in the whole volume, not only at the surface (outer and inner), as demonstrated with CA beads. Consequently, the porosity and pore size distribution changes were observed. There is a lack of data in the literature on similar reactions for a comparison.

### 3.4. Contact Angle Measurements 

**Figure 7:**
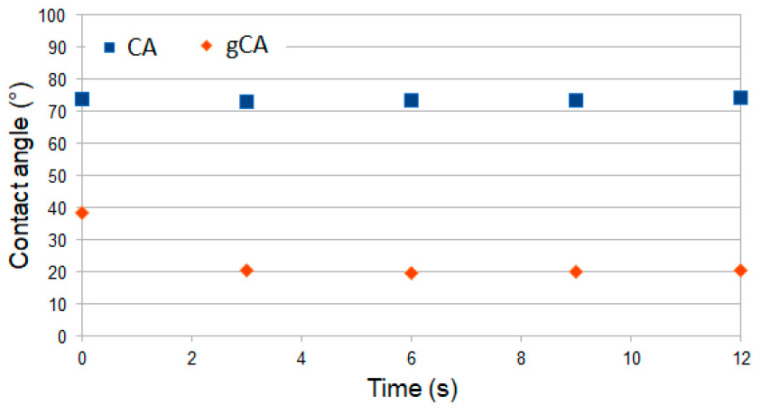
Evolution of contact angle for CA film (CA) and grafted CA film (gCA).

**Figure A2:**
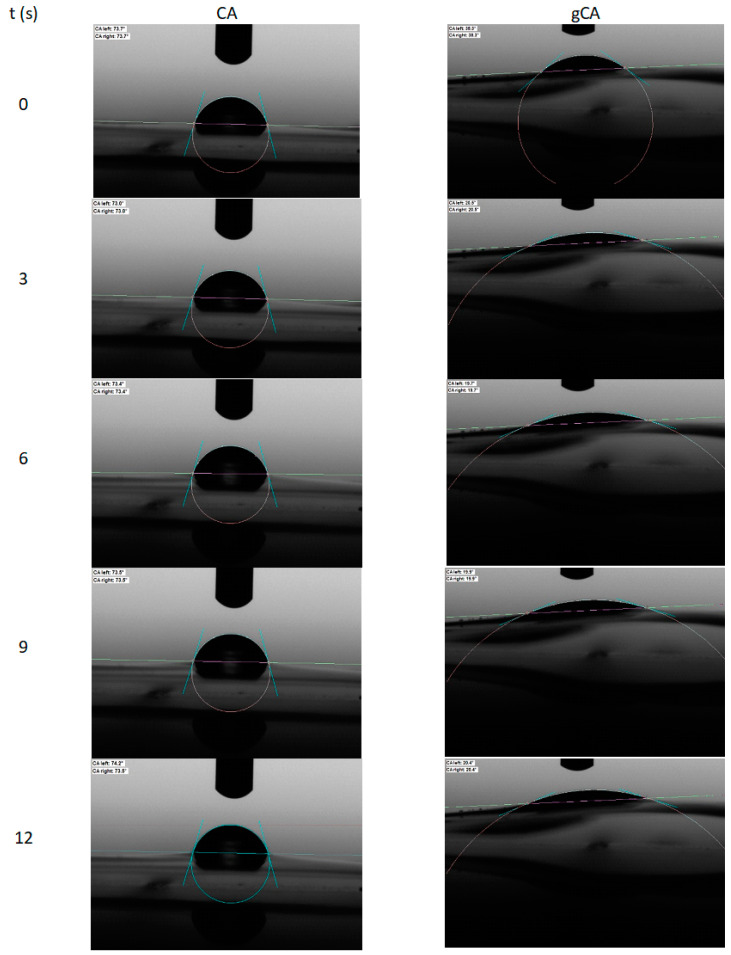
Results of the contact angle measurements for CA film (CA) and grafted CA film (gCA).

Contact angle measurements were performed with cellulose acetate films obtained with the solvent-casting method and dried in air to attain a nonporous structure. The films were further grafted with APDEMS in the same manner as the membranes. The contact angle evolution of pure (CA) and grafted (gCA) film is presented in Figure 7. The images of the water drop in contact with film during 12 s are shown in Figure A2 (Appendix A). The contact angle measurements showed that the treatment increased the material hydrophilicity. The result agrees with FTIR analyses that revealed the presence of unreacted ethoxy groups on the polymer surface. 

### 3.5. Tests in the Cross Filtration Unit

**Figure 8:**
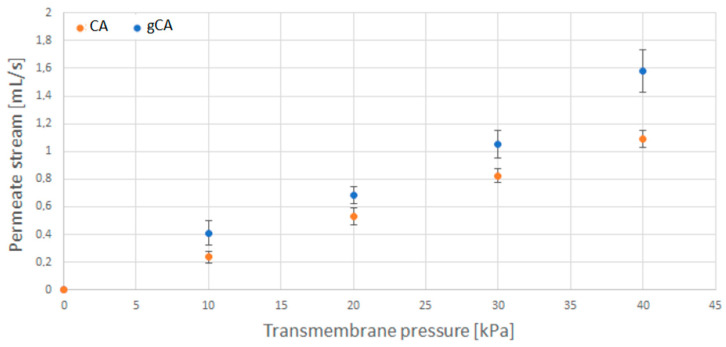
Water permeate stream flow rate as a function of transmembrane pressure for neat (CA) and grafted (gCA) membrane.

The results of water permeate flow rate measurements for neat (CA) and grafted (gCA) membranes are presented in Figure 8. The data revealed an increased permeate flow rate in the modified membrane. This result is a consequence of the more hydrophilic nature of the modified material.

**Figure 9:**
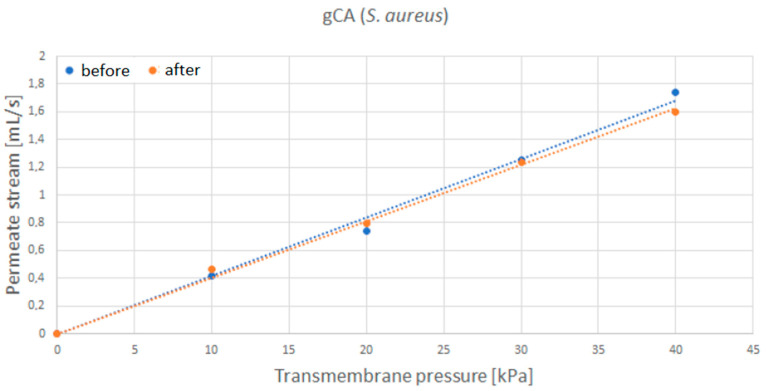
Water permeate flow rate through the grafted membrane (gCA) before and after exposure to *S. aureus*.

**Figure 10:**
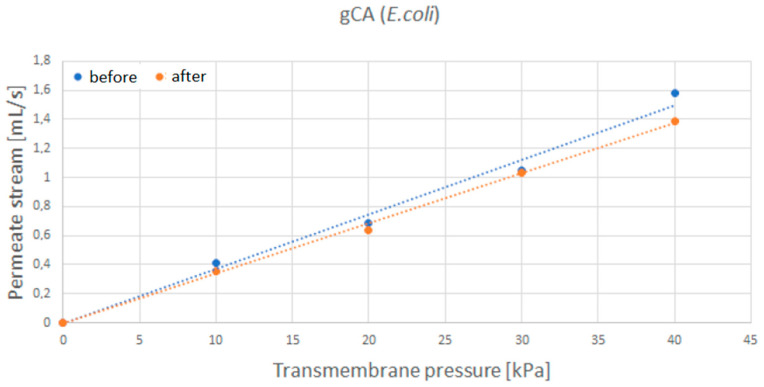
Water permeate flow rate through the grafted membrane (gCA) before and after exposure to *E. coli*.

**Figure A3:**
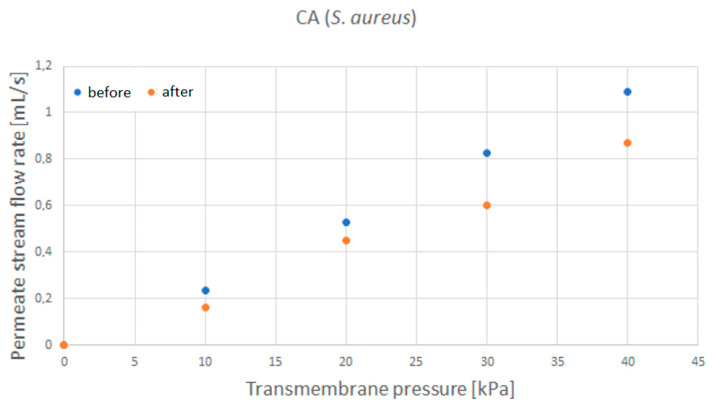
Water permeate flow rate through the neat membrane (CA) before and after exposure to *S. aureus*.

**Figure A4:**
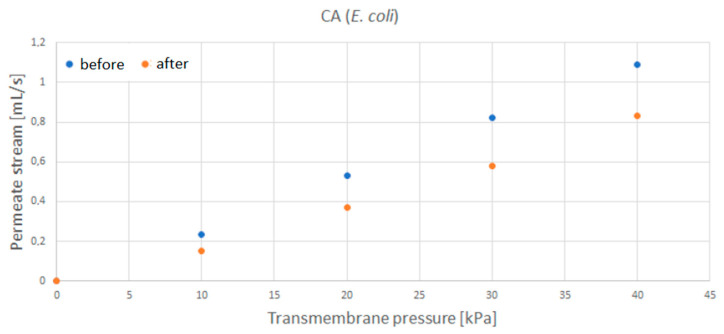
Water permeate flow rate through the neat membrane (CA) before and after exposure to *E. coli*.

After the incubation with *S. aureus*, the water permeate stream flow rate through the grafted membrane only slightly decreased (Figure 9), indicating minor membrane blockage. In the case of *E. coli* (Figure 10), similar results were obtained. The most considerable flow rate decrease of 8% was recorded for the transmembrane pressure of 40 kPa. However, neat membranes showed a more significant flow rate decrease of around 20% for *S. aureus* and 24% for *E. coli* (Figure A3 and Figure A4, respectively, Appendix A).

### 3.6. Evaluation of Bacterial Adhesion to the Membranes

**Figure 11:**
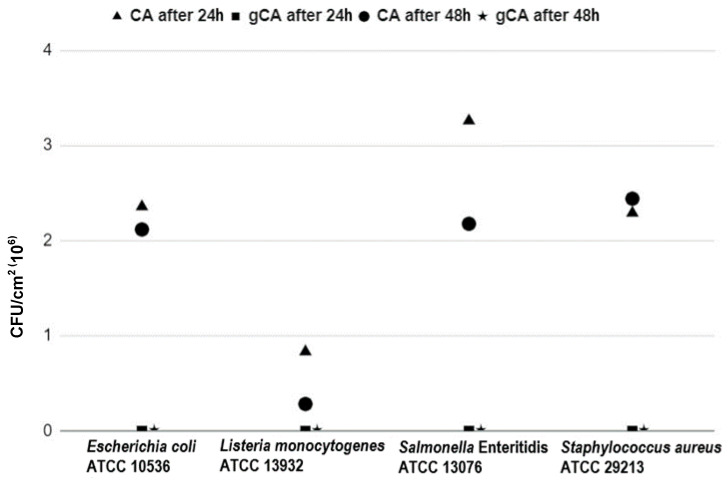
Graphical representation of the number of attached bacteria on cellulose-acetate membranes.

The results of the microbiological investigations are shown in Table 1 and Figure 11. All presented values are mean values obtained from three repetitions. None of the investigated bacterial strains were capable to adhere to the gCA surface after either 24 or 48 h of incubation. In contrast, *E. coli* ATCC 10536 adhered to the CA membranes in a total number of 2.4 × 10^6^ CFU/cm^2^ and 2.1 × 10^6^ CFU/cm^2^ after 24 h and 48 h of incubation, respectively. *L. monocytogenes* ATCC 13932 in a total number of 8.0 × 10^5^ CFU/cm^2^ was detected on CA after 24 h and 3.0 × 10^5^ CFU/cm^2^ after 48 h of incubation. On the CA surface, *S.* Enteritidis attached in a total number of 3.3 × 10^6^ CFU/cm^2^ and 2.2 × 10^6^ CFU/cm^2^ after 24 h and 48 h of incubation, respectively. *S. aureus* ATCC 29213 in a total number of 2.3 × 10^6^ CFU/cm^2^ and 2.4 × 10^6^ CFU/cm^2^ was adhered to CA after 24 and 48 h of incubation, respectively. 

The first step in the microbial contamination of the abiotic materials or colonization of living tissues is the adhesion of bacteria to the surface [39,40,41]. In other words, contamination, colonization, and thus infection, are not possible without successful adherence. Adhesion is conditioned by the type of bacteria, the physical properties of the surface, the availability of nutrients as well as the molecules of the quorum sensing (QS) system [40,41]. Adherence, which goes through a reversible and irreversible phase, can have further consequences in the form of biofilm formation, which then represents a continuous source of pathogenic bacteria causing possible (recurrent) infections in humans and animals, as well as hygienic, sanitary, and technological problems in the food industry, agricultural facilities, and public health [39,40,41]. The production of new materials with physical and chemical properties that disable bacterial adhesion has been imposed as an important and promising strategy to combat biofilms [42,43]. 

## 4. Conclusions

The cellulose acetate membranes were successfully grafted with 3-aminopropyl (diethoxy)methylsilane in supercritical carbon dioxide. Grafting caused a decrease in mean pore diameter from 0.374 µm to 0.321 µm. The porosity changed from 71% to 67.3%. However, the membranes’ functionality was not affected by the modification. Increased polymer hydrophilicity and decreased mean pore diameter resulted in a larger permeate stream flow rate through the modified membrane. Upon a week of incubation with *S. aureus* and *E. coli* cultures, the grafted membranes were characterized by a slight decrease in the permeate stream flow rate, unlike the neat cellulose acetate membranes that were considerably blocked. The grafted membranes showed strong antibiofilm properties in investigations with *S. aureus*, *L. monocytogenes*, *E. coli*, and *S.* Enteritidis. The grafted membranes are suitable for usage in air conditioning systems. Further studies are needed to evaluate the membranes’ performances for particular filtration applications.

## Figures and Tables

**Table 1:** The number of attached bacteria expressed as CFU/cm^2^ on a CA and gCA after 24 h and 48 h of incubation.

Investigated Microorganism	CA 24 h	CA 48 h	gCA 24 h	gCA 48 h
*Escherichia coli* ATCC 10536	2.4 (±0.7) ^1^ × 10^6^	2.1 (±0.3) × 10^6^	0	0
*Listeria monocytogenes* ATCC 13932	8.0 (±0.3) × 10^5^	3.0 (±0.0) × 10^5^	0	0
*Salmonella* Enteritidis ATCC 13076	3.3 (+2.3) × 10^6^	2.2 (+1.5) × 10^6^	0	0
*Staphylococcus aureus* ATCC 29213	2.3 (+0.5) × 10^6^	2.4 (+0.9) × 10^6^	0	0

^1^ SD values are presented in parentheses.

## Appendix A

### The Solvent Casting Method

0.25 g of CA powder were dissolved in 15 mL of acetone by stirring for 2 h. The solution was poured in a Petri dish and left covered overnight for drying.
